# Preparation of Magnetic Nanoliposomes of Sesquiterpene-Rich Fraction from* Cichorium glandulosum* and Its Tissue Distribution in Mice

**DOI:** 10.1155/2018/8549519

**Published:** 2018-10-18

**Authors:** Yuqin Luo, Weijun Yang, Abudujilili Abuduaini, Haji Akber Aisa

**Affiliations:** ^1^Chinese Academy of Sciences Key Laboratory of Plant Resource and Natural Products Chemistry, Xinjiang Technical Institute of Physics and Chemistry, Chinese Academy of Sciences, Urumqi 830011, China; ^2^University of Chinese Academy of Sciences, Beijing 100039, China; ^3^The Institute of Medicine of Xinjiang Uyghur Autonomous Region, Urumqi 830044, China

## Abstract

**Objective:**

To prepare and study the distribution of magnetic nanoliposomes of Sesquiterpene-Rich Fraction from* Cichorium glandulosum* (SRF-MLN) (under magnetic field) in the main organs of mice.

**Methods:**

The SRF-MLN was prepared by ethanol injection-sonication precipitation method. The quality of its pharmaceutical properties was investigated by the active ingredient lactucin. The drug concentration and distribution of lactucin in different tissues and organs including whole blood, liver, heart, spleen, lung, and kidney were evaluated with Sesquiterpene-Rich Fraction from* Cichorium glandulosum* (SRF-LP) as a control.

**Results:**

The prepared SRF-MLN was spherical and monodispersed with an average particle diameter of 65 ± 1 nm, encapsulation efficiency of 91.3%  ± 1.5% (RSD% = 1.5), and drug loading rate of 4.7%  ± 0.3% (RSD% = 0.7). Dispersion coefficient PDI was 0.157 and Zeta potential was −17.5 mV. The lactucin concentration of SRF-MLN after 10 min of intravenous injection in heart, liver, spleen, lung, and kidney was significantly higher than that of SRF-LP group. The AUC_0-12h_ values of liver tissue were obviously higher than other tissues and MRT was significantly prolonged under the action of the magnetic field (*p*<0.01).

**Conclusion:**

The prepared SRF-MLN can change the distribution of drugs in different tissues and organs, prolonging the action time of the drugs in the body, and it has certain specificity under the action of applied magnetic field.

## 1. Background

The liver is an important organ involved in the process of digestion, excretion, detoxification, and immunity. It is prone to all kinds of metabolic and infectious diseases and primary and secondary tumors. There is a high incidence of liver diseases in China. Liver diseases have become common and frequent and could lead to severe liver failure and hepatic encephalopathy, which severely threaten the human health with high morbidity and mortality. Though there have been many drugs used for the treatment of liver diseases, some of them have problems such as lack of specificity, intensive toxic side effects, or instability in the body, which have limited their clinical application. Therefore, exploring effective treatment for liver disease has become a research hotspot.

Hepatic targeted drug delivery system (HTDDS), which effectively delivers the drug to the lesion of the liver, can reduce the dosage and the number of administration times, improve the therapeutic index of the drug, and reduce the adverse reaction. The application of a special carrier is the primary means of liver-targeted drug transport. The magnetic nanocarrier carries the therapeutic agent to the target site with the aid of a magnetic field, which allows the therapeutic agent to concentrate in the target area and reduces the distribution of the drug at other sites, and thereby could enhance the curative effect and reduce the dosage and the side effects of drugs [1–6]. Due to its good biocompatibility, nontoxicity, biodegradability, and good magnetic field response, magnetic nanoliposomes are widely used in the field of biomedicine.

*Cichorium glandulosum *Boiss. et Huet is the Chicory plant in Asteraceae family, which is widely cultivated in Xinjiang, China. There are two species of* Cichorium *genus,* C. glandulosum* Boiss. et Huet and* C. intybus* L., collected in the 2015 edition of the Chinese Pharmacopoeia. Their seeds, aerial parts, and roots have been used in traditional Uyghur medicine for the treatment of jaundice with damp-heat pathogen, stomach pain and low food intake and edema oliguria. They proved to have the functions of clearing away liver-fire and gallbladder-heat, removing stagnated food by using stamachics, and inducing diuresis to reduce edema. Flavonoids, sesquiterpenes, triterpenes, coumarins, alkaloids, sterols, and other active ingredients have been reported in these two plants [7–12]. Our research group has performed the investigations on* Cichorium glandulosum* for more than ten years [13–15]. In the present study, the aerial parts of* C. glandulosum* were extracted, purified by macroporous resin to obtain the effective fraction of* C. glandulosum* total sesquiterpene (SRF) with the protective activity of liver injury by efficacy tracking method. Due to the low SRF yield (about 0.8%), it is difficult for common dosage forms to reach effective plasma concentrations at the target site. In this experiment, SRF was used as a model drug to prepare the magnetic nanoliposomes of* C. glandulosum* total sesquiterpene (SRF-MLN) by ethanol injection-ultrasound precipitation. Under the applied magnetic field, the distribution of SRF-MLN in liver, heart, spleen, lung, kidney, and other tissues and organs was studied after its injection in mice tail vein and also was compared with the liposomes of* C. glandulosum* total sesquiterpene (SRF-LP).

## 2. Materials and Methods

### 2.1. Instruments

The instruments used are Agilent 1260 High Performance Liquid Chromatography (Agilent Technologies, USA), Millipore Simplicity-185 Ultra Water Purifier (Millipore Corporation, USA), JIN92-IIN Ultrasonic Cell Shredder (Ningbo Xinzhi Biotechnology Co., Ltd.), and Nano S90 Laser Particle Size Analyzer (British Malvern).

### 2.2. Materials

Soy lecithin was purchased from German lipoid company; hydrogenated soy lecithin was purchased from Japan Fine Chemicals Co., Ltd.; cholesterol was purchased from China Huixing Biochemical Reagent Co., Ltd.; lactucin control is homemade; methanol and acetonitrile were purchased from Fisher, USA; the remaining reagents are of analytical grade.

### 2.3. Methods

#### 2.3.1. Preparation of SRF-MLN

Soybean lecithin, hydrogenated soybean lecithin, and cholesterol were accurately weighed and put in conical flask, then the total sesquiterpene ethanol solution 10mL was added, ultrasonic Cell Shredder was used for 5 minutes to completely dissolve it, 2 mL of phosphate buffer solution (pH = 7.4) was added, with magnetic stirring (60°C) for 45 min, shaking off ethanol, probe ultrasound (400 W) was used 150 times, and the total sesquiterpene common liposomes (SRF-LP) were obtained. The prepared SRF-LP was injected into a suspension of 2% Tween-80 Fe_3_O_4_ magnetic nanoparticles at a flow rate of 2 mL/min, being shaken at 55°C for 30 min and allowed to stand for 1h to obtain SRF-MLN.

### 2.4. Determination of Average Particle Size, Distribution, and Zeta Potential of SRF-MLN

After appropriate amount of SRF-MLN had been measured and making suspension dilution with water, a laser particle size analyzer was used to determine the size, distribution, and Zeta potential.

### 2.5. Encapsulation Rate and Drug Loading Rate

Precision drawing 3 mL of shaped drug was loaded on the treated SephadexG-50 column (inner diameter 1cm, length 27 cm), eluted with pH 7.4 PBS, the volume flow rate was 1mL/min, and the drug-containing liposomes and free drug were collected. In addition, the same batch of raw drug absolute ethanol solution was diluted with PBS solution (pH 7.4) at a ratio of 1:3. The peak area was measured by injecting 10 *μ*L of HPLC, respectively, and the amount of drug in the liposome and the amount of free drug were calculated.

Encapsulation rate (%) = liposomal drug content/(liposomal drug content + amount of free drug) x 100%

Drug loading rate (%) = liposomal drug content/(liposomal drug content+ accessories amount) x 100%

### 2.6. Drug Determination of Blood and Various Tissues and Organs

#### 2.6.1. Sampling and Administration

Kunming mice 120 (purchased from the Experimental Animal Center of Xinjiang, China), weighing 22~24g, were randomly divided into 18 groups, each group of 5, of which 6 were NS administration groups, 6 were total sesquiterpene liposomes (SRF-LP) administration groups, and the other 6 were total sesquiterpene magnetic nanoliposomes (SRF-MLN) administration groups and the loss area was put in magnetic field (a circular magnet with a diameter of 5 mm and a surface magnetic field of 2500 Gauss was fixed on the bilateral skin surface of mice). SRF, SRF-LP, and SRF-MLN were administered 0.5mL at 20 mg/kg of the same dose of mice tail vein, after administration of 0.083, 0.167, 0.333, 0.5, 1, 2, 4, 6, and 12 h, blood and some organs were collected and animals were sacrificed. The amount of lactucin was measured by HPLC.

#### 2.6.2. Determination of Lactucin by HPLC

Lactucin was determined by HPLC as follows: column molecular: Kromasil 100-5 C_18_ column (4.6mm × 250mm, 5 *μ*m); guard column: Fusion-RP 4 × 3.0mm; mobile phase: methanol (A) − 0.2% formic acid (B) gradient elution (0~30 min, 15%~28% A; 30~40min, 28%~15% A); wavelength of detection: 256 nm; flow rate: 1mL/min; column temperature: 35°C; injection volume: 20 *μ*L; theoretical plate height: not less than 3000 degrees of lactucin.

The chromatograms of blank whole blood and tissue extracts showed no other impurity peaks at the peak of the drug, indicating no interference with the drug assay.

The linear regression equation was A = 30324C + 840.15 (r = 0.9999) with the peak area A as the ordinate and the concentration C as the abscissa. The linear range was 1.0−240.5 *μ*g • mL^−1^. The recoveries of three high, middle, and low concentrations of lactucin were (98.1 ± 2.3)%, (99.8 ± 2.7)%, and (103.3 ± 2.2)%, respectively. The repeatability and stability were 1.8% and 2.4%, respectively.

#### 2.6.3. Sample Processing

*Blood Collection*. By using an anesthetic vaporizer (RC-2 Rodent Circuit Controller, VetEquip, Pleasanton, CA), mice were anesthetized by isoflurane inhalation in an induction box (15cm*∗*10cm*∗*10cm Chamber, product no. V100, RWD Life Science Co., Ltd, China) at 2.5% to 3% and maintained at 1% to 2% isoflurane through a nosecone (Bubble Tubing Nosecone, small to medium mouse [9 mm], product no. 921609, VetEquip) on a Bain nonrebreathing circuit (Pedi-Bain 45-in., product no. 921410, VetEquip). The mice were confirmed to be at a surgical plane of anesthesia through the lack of a pedal reflex (no response to a firm toe pinch).

1. The left thumb and the index finger grasp the ears of the mouse and the skin behind the neck, and the little finger fixes the tail.
2. The middle finger gently presses the left forelimb of the mouse on the sternal heart, and the ring finger presses on the abdomen. The thumb shakes and gently presses around the eyeball to make the eyeball congestion.
3. Use the elbow to pick up the eyeball.
4. Tilt the direction of the thumb and forefinger as needed to allow blood to flow vertically from the eyelid at different speeds into the centrifuge tube.
5. Simultaneously press the heart part of the mouse with the left middle finger to speed up the pumping speed of the heart.
6. When the blood is exhausted, the mice are sacrificed by dislocation.

*Tissue Collection and Processing*. Heart, liver, spleen, lung, and kidney were cut with accurate amount of 0.1 g, respectively. We added saline (w/v = 1:3), ultrasonic cell mill homogenate, each sample for 3 min, centrifuged for 10 min, took supernatant 200 *μ*L, added methanol 1.0 mL, dense plug, vortex mixed for 3 min, centrifuged in high-speed centrifuge (12000 rpm) for 15 min, and the supernatant in the 40°C water bath nitrogen flow, dried with 100 *μ*L of methanol, and took 20 *μ*L of sample.

The examination methods of all samples were mentioned previously in Section 2.5.

### 2.7. Statistical Analysis

All data were analyzed with SPSS (version 4.0.100.1124; IBM Corporation, Armonk, NY, USA) software and were shown as mean values ± SD. Statistical comparisons were performed to determine group difference through ANOVA. Significant difference between two groups was evaluated by the Student's* t*-test. Statistical significance was set as follows:* P*, 0.05 and* P*, 0.01.

## 3. Results

### 3.1. Physical and Chemical Properties of SRF-MLN

**Figure 1:**
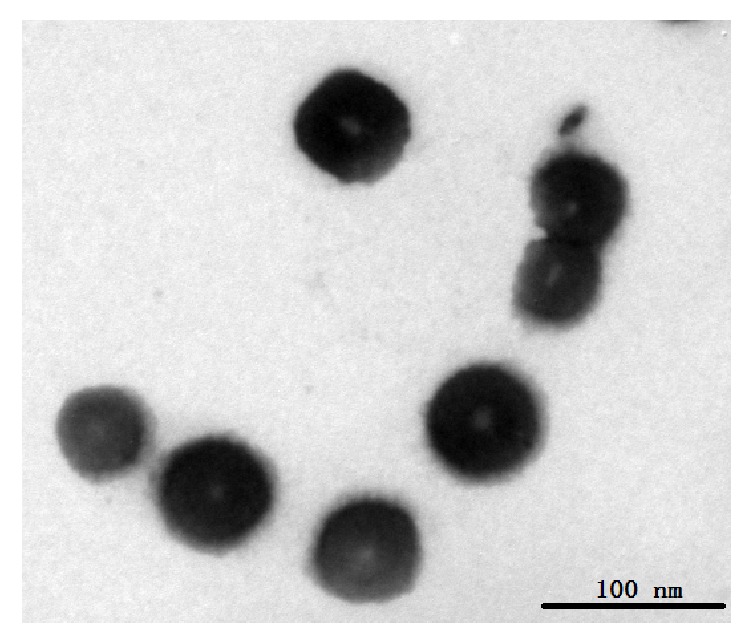
Transmission electron microscopy of SRF-MLN.

SRF-MLN solution was translucent and the average particle size was (65±1nm), dispersion coefficient PDI was 0.157, Zeta potential was −17.5 mV, encapsulation efficiency was 91.3%  ± 1.5% (RSD% = 0.7), and loading was 4.7%  ± 0.3%. The morphology of SRF-MLN observed by transmission electron microscope (TEM) showed that the center of SRF-MLN was obviously wrapped with magnetic nanoparticles and in spherical and monodisperse distribution, as shown in Figure 1.

### 3.2. Tissue Distribution of Drugs in the Body

#### 3.2.1. Standard Curve and Linear Range

0.1 g of each tissue, homogenized with physiological saline, was added to the working fluid 20 *μ*L, equivalent to the plasma concentrations of 0.02, 0.05, 0.1, 0.5, 1.0, and 3.0 *μ*g/mL of the tissue standard solution. According to the above sample processing methods, they were measured by HPLC and linear regression analysis was carried out with the peak area (Y) of the standard as the ordinate and the concentration (C) as the abscissa. The results are shown in Table 1.

#### 3.2.2. Tissue Distribution of Drug in Mice

**Figure 2:**
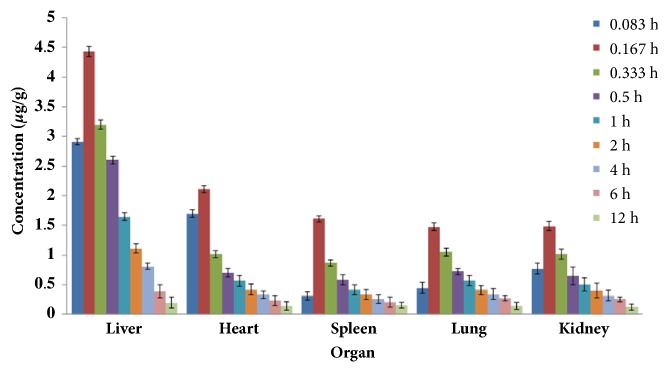
SRF-LP distribution in various organs of mice.

**Figure 3:**
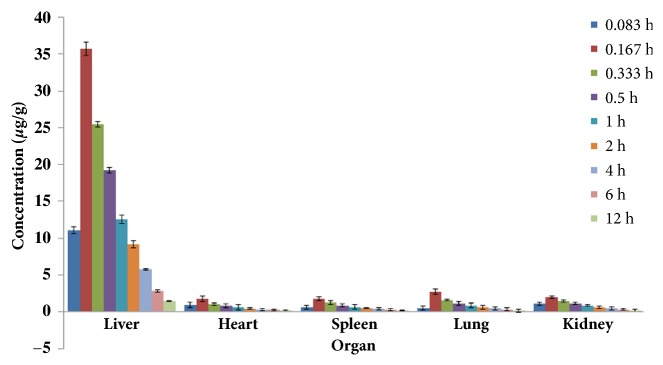
SRF-MLN distribution in various organs of mice.

The amount of drug (*μ*g) per gram tissue was calculated based on the determination of the concentration of the drug in the tissue, see Figures 2 and 3. The area under the curve (AUC_0-12_) and mean residence time (MRT) were calculated by the trapezoidal area method according to the time-concentration changes in the organs and tissues. The results are shown in Table 2.

It can be seen from Figures 2 and 3 that the content of lactucin in the liver, heart, spleen, lung, and kidney of SRF-MLN group was higher than that of SRF-LP group.

It can be seen from Table 2 that both AUC_0-12h_ and MRT were higher in SRT-MLN mice than those in SRF-LP mice. AUC_0-12h_ and MRT in the liver in SRF-MLN group were significantly greater than those in other organizations compared with the SRF-LPN group, and the difference was significant (*p* < 0.01).

## 4. Discussions

In this study, the magnetic nanoliposomes were prepared by ethanol injection combined with ultrasound precipitation using the Sesquiterpene-Rich Fraction from* C. glandulosum* (SRF) which showed protective activity against liver injury. The liposomes were uniform spherical particles and the particle size was less than 100 nm. After the SRF was made into magnetic nanoliposomes, it was conducive for the active ingredient to be delivered targeted to the lesion site, which reduced the distribution of the drug in other sites and the side effects of the drug.

The results of* in vivo* distribution showed that with the change of administration time, the content of lactucin in different dosage forms showed the same trend in heart, liver, spleen, lung, and kidney. The content of lactucin gradually increased in SRF-MLN group than that in SRF-LP group, and the content increased in the liver was particularly notable. It showed that the prepared SRF-MLN in the animal body had good magnetic response under the action of the magnetic field, which could significantly improve the distribution of the effective component of* C. glandulosum* in the tissue of mice, especially in the liver. Prepared liver-targeted magnetic liposomes could delay the elimination of lactucin in body to improve the bioavailability with a certain sustained release.

However, a series of in-depth studies should be further carried out for the magnetic nanoliposomes to be applied clinically. Although there are still many problems to be solved regarding magnetic targeted drug delivery, the research contents of this project can provide some guidance and demonstrations for the preparation and study of novel liver-targeted drug delivery carriers in Uyghur medicine.

## Figures and Tables

**Table 1:** The standard curve of lactucin in different groups.

tissue	standard curve	R^2^
blood	Y = 38.533X + 0.765	0.9994
liver	Y = 39.248X + 0.705	0.9985
heart	Y = 39.212X + 0.298	0.9939
spleen	Y = 40.261X + 0.439	0.9991
lung	Y = 40.256X − 0.231	0.996
kidney	Y = 38.382X + 0.301	0.9968

**Table 2:** AUC_0-12h_ and MRT values in tissues.

tissues	SRF-LP	SRF-MLN	SRF-LP	SRF-MLN
blood	2.3769±0.4281	4.0819±3.2735	4.4439±0.6556	5.0875±0.6771
liver	3.0093±0.2127	32.063±4.5521	3.7804±0.1488	10.049±1.3722
heart	1.5428±0.1129	1.6408±0.2227	2.5809±0.0876	3.0996±0.4522
spleen	0.7503±0.1516	1.7894±0.1995	2.9247±0.3448	7.5432±0.5365
lung	0.824±0.1228	2.2061±0.2236	2.6645±0.2167	7.2248±0.6571
kidney	0.7176±0.1205	1.9651±0.2788	2.8994±0.0891	5.7854±0.4559

## Data Availability

The data used to support the findings of this study are available from the corresponding author upon request.

## Abbreviations

SRF: Sesquiterpene-Rich Fraction from* Cichorium glandulosum*
SRF-LP: Liposomes of Sesquiterpene-Rich Fraction from* Cichorium glandulosum*
SRF-MLN: Magnetic nanoliposomes of Sesquiterpene-Rich Fraction from* Cichorium glandulosum*
TE: Targeting efficiency
RTE: Relative targeting efficiency
TI: Targeting index
MRT: Mean residence time
RSD: Relative standard deviation
HTDDS: Hepatic targeted drug delivery system
AUC: Area under curve.
